# Randomised pilot and feasibility trial of a group intervention for men who perpetrate intimate partner violence against women

**DOI:** 10.1186/s12889-024-18640-5

**Published:** 2024-04-27

**Authors:** Helen Cramer, Daisy M. Gaunt, Rebekah Shallcross, Lis Bates, Rebecca Kandiyali, LynnMarie Sardinha, Caoimhe T. Rice, Mei-See Man, Gene Feder, Tim J. Peters, Karen Morgan

**Affiliations:** 1 Centre for Academic Primary Care, Population Health Sciences, Bristol Medical School, University of Bristol, Bristol, UK; 2 Bristol Trials Centre, Population Health Sciences, Bristol Medical School, University of Bristol, Bristol, UK; 3 Connect Centre, University of Central Lancashire (UCLan), Preston, UK; 4 Health Economics Bristol, Population Health Sciences, Bristol Medical School, University of Bristol, Bristol, UK

**Keywords:** Domestic abuse, Domestic abuse perpetrator programme, Domestic violence perpetrator programme, Batterer program, Male perpetrator, Female victim survivor, Complex intervention

## Abstract

**Background:**

There is a need for robust evidence on the effectiveness and cost-effectiveness of domestic abuse perpetrator programmes in reducing abusive behaviour and improving wellbeing for victim/survivors. While any randomised controlled trial can present difficulties in terms of recruitment and retention, conducting such a trial with domestic abuse perpetrators is particularly challenging. This paper reports the pilot and feasibility trial of a voluntary domestic abuse perpetrator group programme in the United Kingdom.

**Methods:**

This was a pragmatic individually randomised pilot and feasibility trial with an integrated qualitative study in one site (covering three local-authority areas) in England. Male perpetrators were randomised to either the intervention or usual care. The intervention was a 23-week group programme for male perpetrators in heterosexual relationships, with an average of three one-to-one sessions, and one-to-one support for female current- or ex-partners delivered by third sector organisations. There was no active control treatment for men, and partners of control men were signposted towards domestic abuse support services. Data were collected at three-monthly intervals for nine months from male and female participants. The main objectives assessed were recruitment, randomisation, retention, data completeness, fidelity to the intervention model, and acceptability of the trial design.

**Results:**

This study recruited 36 men (22 randomly allocated to attend the intervention group programme, 14 to usual care), and 15 current- or ex-partners (39% of eligible partners). Retention and completeness of data were high: 67% of male (24/36), and 80% (12/15) of female participants completed the self-reported questionnaire at nine months. A framework for assessing fidelity to the intervention was developed. In interviews, men who completed all or most of the intervention gave positive feedback and reported changes in their own behaviour. Partners were also largely supportive of the trial and were positive about the intervention. Participants who were not allocated to the intervention group reported feeling disappointed but understood the rationale for the trial.

**Conclusions:**

It was feasible to recruit, randomise and retain male perpetrators and female victim/survivors of abuse and collect self-reported outcome data. Participants were engaged in the intervention and reported positive benefits. The trial design was seen as acceptable.

**Trial registration:**

ISRCTN71797549, submitted 03/08/2017, retrospectively registered 27/05/2022.

**Supplementary Information:**

The online version contains supplementary material available at 10.1186/s12889-024-18640-5.

## Introduction

### Background

Domestic abuse, including intimate partner violence (IPV), is a violation of human rights that damages physical and mental health and the wellbeing of victim/survivors and their families. The United Nations Declaration on the elimination of violence against women [1] defines violence against women as *‘*any act of gender-based violence that results in, or is likely to result in, physical, sexual or psychological harm or suffering to women, including threats of such acts, coercion or arbitrary deprivation of liberty, whether occurring in public or in private life’. Of women aged 15 years and older who have ever been in a relationship, 26% have been subjected to physical and/or sexual violence from an intimate partner in their lifetime [2]. In England and Wales in the year ending March 2022, an estimated 7.9% of women aged 16–59 experienced domestic abuse, and 72% of victims of domestic homicide were female [3]. Men also experience domestic abuse, but there are differences in the types of abuse (fewer experience sexual violence, or coercive and controlling behaviours), amount of abuse, severity of abuse and its impact [4]. In 2017 the cost of domestic abuse in England and Wales was approximately £66billion through physical injuries and emotional harm, time off work and costs associated with health services, the criminal justice system, social welfare and the provision of victim services [5]. Responses to IPV include: support for victim/survivors, health service interventions, legal and judicial reforms [6]. Domestic Abuse Perpetrator Programmes (DAPPs) emerged in the 1980s to address IPV perpetrators’ behaviour [7]. DAPPs are aimed at perpetrators who are considered at the mild to moderate end of abusive behaviours and the programmes draw on various approaches: psychoeducational models, cognitive behavioural therapy (CBT), and the Duluth model which includes a coordinated community response [8, 9]. Respect is the UK’s accreditation organisation for work with perpetrators, and Respect-accredited DAPPs involve group work for the perpetrators, safety planning and support work with current- or ex-partners, and information-sharing within the programme and other relevant agencies [10, 11]. Evidence of the efficacy of DAPPs is mixed although some randomised trials have reported reductions in abuse for DAPPs [12–16] and there have been positive benefits reported from non-randomised evaluations and reviews [17–19]. Gondolf and colleagues found that well-established perpetrator programmes across four cities in the United States with a population mandated to attend did appear to reduce physical assault [20]. Other studies have not found evidence of change in abusive behaviour for DAPPs [21–23], or only slight reductions in abuse [24]. However, the methodological limitations of evaluations leaves substantial uncertainty about the effectiveness of DAPPs [25]. Gondolf [26] noted that randomised controlled trials are challenging for IPV in mandated populations, especially in situations where allocation to a control group might be overruled by judges. Other methodological issues include an over-reliance on police incident report data, no victim/survivor data, poorly measured outcomes, heterogeneous populations, high attrition rates and a reliance on short duration of follow-up [10, 27–32]. Although there is some strong scepticism in the field as to whether randomised controlled trials are the best way to evaluate perpetrator programmes, especially given the potential challenges of capturing the complex ambiguities of changing abusive behaviours, [33–35] we decided it was still a useful exercise to attempt. Addressing some of the evidence gaps and methodological shortcomings of previous evaluations, we piloted an individually randomised controlled trial of a community-based intervention in the UK. The aim of this pilot and feasibility trial was to assess the feasibility and acceptability of a future definitive randomised controlled trial.

### Objectives

The primary objective was to assess recruitment, randomisation, and retention of men and their current- or ex- partners (hereafter referred to as (ex)partners).

Secondary objectives were to: 1) assess questionnaire completion; 2) develop a fidelity framework for intervention; 3) identify and pilot the collection of resource-use data and the associated unit costs for cost-effectiveness analysis; 4) determine acceptability of the intervention to perpetrators, associated victim/survivors and staff; 5) assess the willingness and risks faced by female (ex)partners to be involved in the pilot trial; 6) explore ways of improving retention in both the intervention and control arms for male and female participants; and 7) assess the mechanisms of support and supervision needed for those involved in the delivery of the intervention.

We were also interested in determining the most appropriate self-reported outcome measures to capture change in abuse, and whether the abuse reported by either the (ex)partners or by the male perpetrators (or both) should be the primary outcome in the definitive trial.

## Methods

### Trial design

A pilot and feasibility trial of a pragmatic, community-based, parallel, individually randomised controlled trial with a 2:1 intervention to usual care allocation ratio. The group intervention was delivered by a Respect-accredited organisation that specialised in working with IPV perpetrators.

### Participants

The Reaching Everyone Programme of Research On Violence In diverse Domestic Environments (REPROVIDE) was a research programme which includes the REPROVIDE pilot and feasibility trial which recruited men who lived in three local-authority areas in England: Bristol, North Somerset and South Gloucestershire. Men could self-refer or be referred by any service working with them. The research team would call the potential participant to explain the trial and confirm eligibility (Table 1). Potential participants were invited to an assessment with a researcher and the intervention programme coordinator. At the assessment, they would be asked about their abusive behaviour to ascertain levels of risk, assessed for their acknowledgement of abusive behaviour and motivation to change. The assessment of risk would be judged mainly by the programme coordinator based on what the man disclosed, any information a referrer gave and if the man’s participation was thought to place the (ex)partner at greater risk. If eligible for the trial, they gave informed consent, completed a baseline questionnaire, and were then randomly allocated. Up to two female (ex)partners of the recruited male participants were contacted. All (ex)partners who met the inclusion criteria (Table 1) were invited to participate in the trial.

**Table 1:** Inclusion and exclusion criteria for men and (ex)partners

Inclusion criteria	Exclusion criteria
**Men**	
Aged 21 or over	Did not have a current or previous female partner
Used (and acknowledged) abusive behaviour in his current or past relationships with women	Who had been court-mandated to attend a DAPP
Ability to read and complete outcome questionnaires (with or without the support of a researcher)	Deemed by the programme coordinator as high risk or unable/unwilling to engage in the intervention
	Could not speak English well enough to give informed consent or take part in a group
	A diagnosis of mental illness (such as active psychosis) that would prevent them engaging with the programme
	Unstable substance or alcohol use
**Partners**	
Female partners or ex-partners of men using violence/abuse in their relationships	Participants who cannot understand English sufficiently well to give informed consent and to complete the questionnaires
> 18 years	Women who are deemed by the domestic abuse support worker to be put at greater risk if they take part in the trial
Ability to read and complete outcome questionnaires	Women who are incapacitated by substance abuse or serious mental illness at time of seeking consent

### Intervention

The intervention consisted of a 23-week DAPP with a planned average of three additional one-to-one sessions. The men’s group was run by two facilitators (usually one male and one female). The facilitators had a range of professional backgrounds (psychiatric inpatient, probation and counselling) and experience. The facilitators received one week’s training on the REPROVIDE intervention, three out of four had prior training on DAPPs, the other facilitator did not. The group sessions followed a manual and lasted around 2.5 h. Participants were removed if there was a high level of concern for the safety of their (ex)partner or facilitators. The group intervention had a rolling-intake with new men joining approximately every five weeks. The second intervention group began once enough men (e.g. 7 to 8) were taking part in the first group and at the end of the trial, as numbers diminished, the two groups merged. A monthly relapse prevention group (RPG) was available at the end of the programme for six months.

The programme combined methods: feminist, Duluth, CBT, psychoeducation, and multiagency information-sharing to hold men accountable for their actions (Table 2). The group programme and accompanying manual were developed following a modified two-stage Delphi consensus process [36], by two consultants from Respect [11]. (Ex)partners of men allocated to the intervention were offered weekly one-to-one support from a women’s support worker and there were ongoing risk assessments and multi-agency information sharing where appropriate. Support for (ex)partners continued alongside the men’s group programme and for up to six-months after the end of the programme, in parallel with the men’s RPG. If a man stopped engaging with the trial, (ex)partners would continue to be supported.

**Table 2:** Key intervention group programme processes

Safety planning in the early stages
Work with men to increase capacity to ‘straight talk’/ explore denial
Educational work e.g., widen men’s definition of abuse
Developing a critical awareness of attitudes, beliefs and expectations that support the use of violence and abuse (CBT)
Building empathy for victims (e.g., through role play)
Identifying and practicing alternative behaviour

The control treatment was usual care, and at the time of the pilot there were no other DAPPs running in the recruitment areas. (Ex)partners of men in the control group were signposted towards local women’s domestic abuse support services.

### Public and patient involvement

Public and Patient Involvement (PPI) groups were active throughout the research. A group of women (6–7) who had experienced IPV and a separate group of men (1–4) who had attended a DAPP were consulted at approximately four-monthly intervals. The PPI groups were consulted on the design of the research and the intervention, including questionnaire content and which validated quality of life measure to use, recruitment materials and terminology.

### Outcomes

The pre-specified progression criteria, based on the primary objective, determined whether to proceed with the definitive trial. These were: recruitment of 36 men within nine months and/or a steady state recruitment rate that is consistent with this aim; follow-up of male participants of 0.6 (95% confidence interval 0.4 to 0.8) at nine-months; and follow-up of female (ex)partners of 0.5 (95% confidence interval 0.4 to 0.7) at nine-months. Data were collected from participants by self-report questionnaire on abuse severity and impact (IMPACT toolkit, client and partner versions modified for trial purposes, [37]); mental health symptom severity (depression PHQ-9, [38, 39]); anxiety (GAD-7, [40, 41]); post-traumatic stress disorder (PCL-5, [42]); physical health and health related quality of life (EQ-5D-5L, [43]); short form health survey (SF-12v2, [44]); alcohol use (AUDIT, [45]); drug use (DUDIT, [46]); gambling (NODS-CLiP, [47]). Data were collected at baseline, three-, six- and nine-months (post-randomisation for males, post-recruitment for females), and these data were used to assess questionnaire completion (objective 1). Female (ex)partners were also asked to complete outcomes on their children’s health and wellbeing (KIDSCREENS –10p, [48]). Police incident and crime data for 12-months prior- and post-randomisation were collected for male participants from the police force’s system.

A framework for assessing fidelity to the model (objective 2) was developed using qualitative data from observations and video recordings of the group sessions, researcher fieldnotes of the group facilitator training, focus groups and interviews with facilitators.

The focus of the economic analysis was to identify and pilot methods of data collection of resource-use data and the associated unit costs for cost-effectiveness analysis (objective 3). To inform the identification of relevant measures, we sought input from the female PPI group and other researchers with knowledge of social work, third-sector agencies working with either men or their (ex)partners, and the criminal justice system.

Acceptability of the intervention and trial design (objective 4) was assessed using qualitative data from semi-structured interviews with male and female participants from both trial arms, facilitators, referrers and service commissioners. Intervention feasibility was assessed through intervention engagement and retention, whether intervention delivery was possible in a timely manner and whether the training was possible. The feasibility of identifying and recruiting eligible participants was assessed using qualitative data from semi-structured interviews with referring staff and participants and researcher recruitment fieldnotes. Intervention engagement was captured through group attendance registers, group case notes, and individual case notes, and data from the women’s safety worker on frequency and type of contact. Data were gathered through interviews and (Serious) Adverse Events ((S)AEs)) reports to assess the willingness to engage, and risks faced by (ex)partners to be involved in the pilot trial (objective 5). To assess how to improve retention (objective 6), we spoke to the PPI group and considered how to implement data collection. To review the mechanisms of support and supervision needed for those involved in the delivery of the intervention (objective 7) we regularly met with delivery site staff and asked about the support and supervision they received. Feedback on intervention training, intervention delivery and how the intervention differed to usual practice was sought in interviews and an end-of-trial focus group. In consultation with our programme advisory committees and trial methodologists from Bristol Trials Centre, we assessed the suitability and statistical validity of potential primary outcome measures. For an overview of the intervention and measures please also see the logic model (Additional file 1).

### Sample size

This pilot trial was originally designed to recruit 48 men within six-months, but this was changed to 36 men within nine-months, which would allow reasonable precision for estimating follow-up rates (a key progression criterion). This alteration followed from the change in randomisation ratio from 1:1 to 2:1, keeping the maximum number of men allocated to the intervention at 24 and reducing to 12 the number allocated to usual care. The longer duration for recruitment allowed additional time to recruit facilitators, explain the trial to potential referrers, and promote and randomise sufficient men to form intervention treatment groups (Table 3).

**Table 3:** Amendments

Amendment	Date approved	Approved by
Randomisation ratio amended from 1:1 to 2:1	5 September 2017	Research Ethics Committee (REC)
Travel expenses for low-waged/unemployed men attending the assessments and intervention groups	5 September 2017	REC
Final follow-up of male participants and female (ex)partners amended from one-year to nine-months	5 September 2017	REC
A one-page document with information for professionals added to aid recruitment	5 September 2017	REC
ICECAP-A added to outcome measures collected at nine-month follow-up only	21 February 2018	REC
Recruitment amended from 48 men within six-months to 36 men within nine-months	21 February 2018	REC
Randomisation stratification variable removed: severity of abuse	13 March 2017	Trial team, intervention provider and member of the programme executive group

### Randomisation

Randomisation was conducted online by the researcher in the presence of the man and programme coordinator via a computer programme created by the Bristol Trials Centre. All were immediately informed of the allocation. A minimisation procedure with a probabilistic component was used. Minimisation factors were age (under, or over, 30) and whether men were still in a relationship with the abused partner (living with partner; not living with partner but still in a relationship with partner; separated and not living with partner). Severity of abuse was removed as a minimisation factor as it was felt to be too challenging to ascertain at recruitment. Initially, the randomisation was at a ratio of 1:1. However, following difficulties in recruiting at least in part due to referring services wanting more certainty that their clients would be allocated a place on the intervention, this was changed to a 2:1 ratio in favour of the intervention group (Table 3).

### Methods of analysis

All data on recruitment, randomisation and retention are reported in line with CONSORT guidelines. Descriptive statistics are used to summarise all baseline data, and completion of outcome measures by both trial groups. All interview recordings were transcribed, and qualitative data were analysed using a thematic framework based on the pilot trial objectives. For extra detail please see the coding framework used for the thematic analysis (Additional file 2).

## Results

### Recruitment and retention

**Fig. 1:**
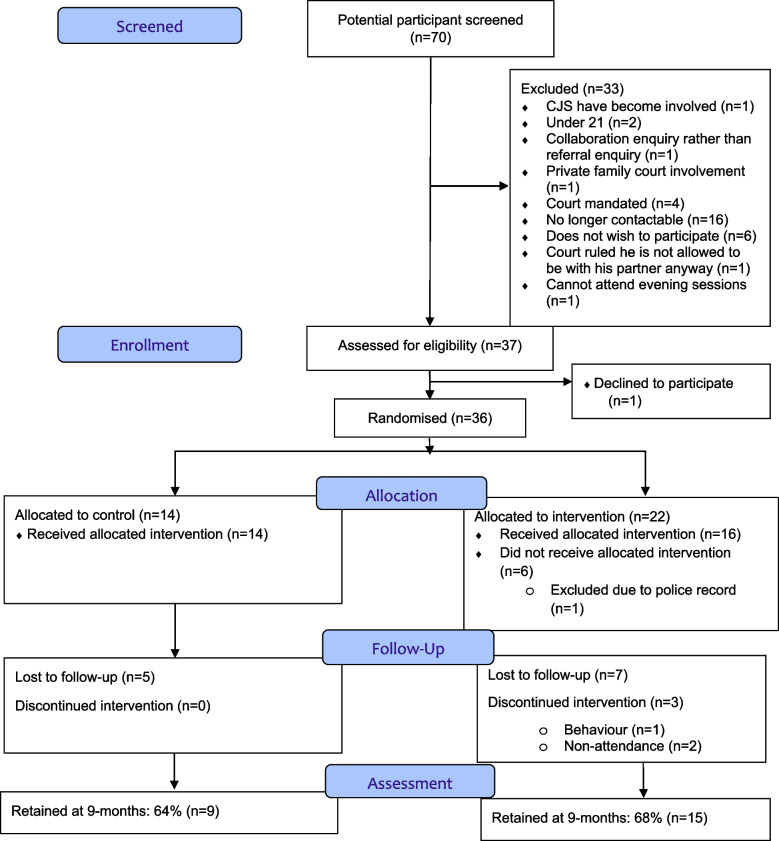
CONSORT chart for male participants

**Fig. 2:**
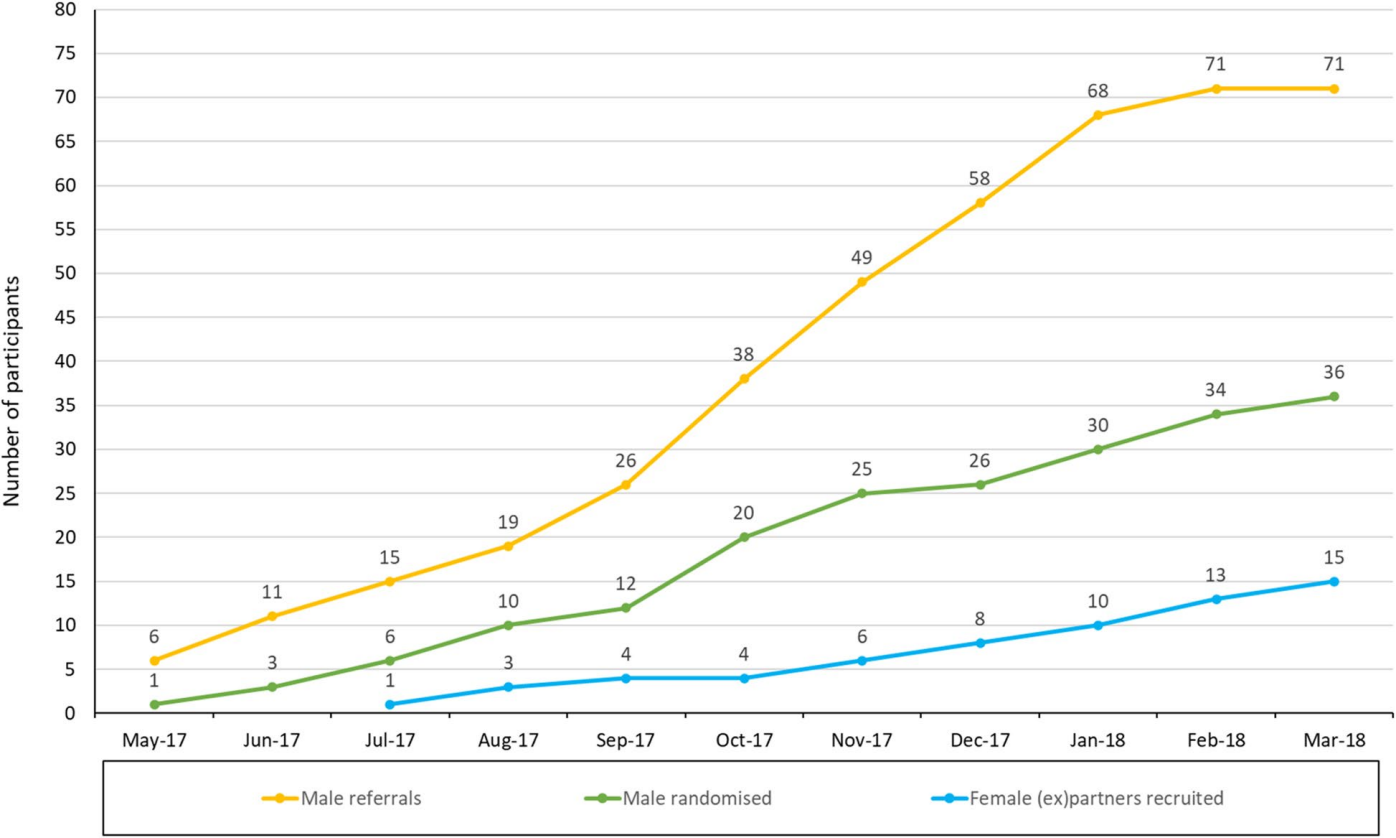
Referral and recruitment of male participants, and female (ex)partners

**Fig. 3:**
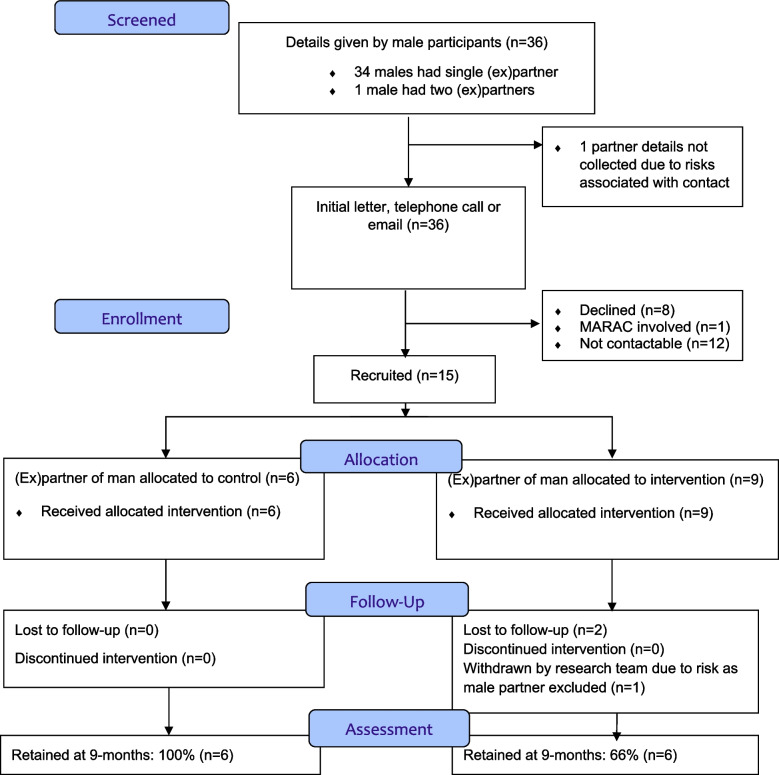
CONSORT chart for female (ex)partners

Participants were recruited between 25th May 2017 and 7th March 2018. A total of 36 men (22 intervention, 14 control) were randomised in less than 11 months (Figs. 1 and 2). Fifteen female (ex)partners were recruited into the trial (39% of partners, 2 (ex)partners of the same male participant) (Figs. 2 and 3).

Referrals came from social work and children’s services, counselling and health services, police and probation, legal services, substance abuse and women’s IPV services. Self-referrals often came to the research team via the national Respect helpline (male perpetrators and victims) or through the DAPP delivery organisation. Promotion to achieve recruitment targets was challenging and twice as many referrals were needed as planned to recruit the men required (Fig. 2). Prior to the pilot trial, no group DAPPs had been operating in the area for several years and so awareness of the service and trial had to be raised. There was some resistance from some potential referrers to interventions focused on perpetrators rather than just victim/survivors, or to a randomised design where participants were not guaranteed a place on a programme. Changing the randomisation ratio from 1:1 to 2:1 ratio in favour of the intervention helped. Reasons given by men for wanting to join the trial were largely focused on their desire to receive the intervention, including: recognising an ‘anger problem’; wanting to be a better parent; wanting to increase the likelihood of child contact (in cases of separated families); and fears that their partner would leave them.

The pre-specified progression criteria to the definitive trial were achieved for both men and (ex)partners at nine-months. Twenty-four (67%, 95% CI 49% to 81%) male participants and 12 (80%, 95% CI 82% to 96%) female (ex)partners were retained to the end of the trial (Figs. 1 and 3).

### Baseline data

The demographic and clinical characteristics of men and their (ex)partners in the intervention and control are reported in Tables 4 and 5. Mean age for men in the intervention group was 38.0 compared with 49.9 for control men; mean age for partners and (ex)partners in the intervention and control groups was 44.1 and 42.8 respectively. Most participants had children.

**Table 4:** Baseline socio-demographic and clinical characteristics of male participants

	**Intervention** ***N* = 22** **n (%)**	**Control** ***N* = 14** **n (%)**
**Mean Age (standard deviation, SD)**	38.0 (11.3)	39.9 (11.1)
**Sexuality**		
Heterosexual/Straight	19/20 (95%)	14 (100%)
**Ethnicity**		
White	20 (91%)	11 (79%)
Mixed / Multiple ethnic group	0	2 (14%)
Asian or Asian British	1 (5%)	1 (7%)
Black /African / Caribbean / Black British)	1 (5%)	0
**Religion**		
No religion	11 (50%)	8 (57%)
Christian	8 (36%)	5 (36%)
Muslim	1 (5%)	1 (7%)
Sikh	1 (5%)	0
Prefer not to say	1 (5%)	0
**Educational Qualifications**		
No formal qualifications	3 (14%)	2 (14%)
Other qualifications	1 (5%)	0
O-levels, GC(S)Es, NVQs 1–3	12 (55%)	7 (50%)
NVQs 4–5, HND	1 (5%)	1 (7%)
Degree or higher	5 (23%)	4 (29%)
**Employment Status**		
Employed	11 (50%)	9 (64%)
Looking after your home/family	4 (18%)	1 (7%)
Unable to work due to long term sickness	1 (5%)	2 (14%)
Retired from paid work	3 (14%)	0 (0%)
Other	3 (14%)	2 (14%)
**Household Income**		
Up to £5000	2 (9%)	1 (7%)
£5,000 up to £11,999	3 (14%)	1 (7%)
£12,000 up to £21,999	5 (23%)	3 (21%)
£22,000 up to £37,999	4 (18%)	2 (14%)
£38,000 up to £71,999	2 (9%)	4 (29%)
£72,000 and above	1 (5%)	2 (14%)
Prefer not to say/do not know	5 (23%)	1 (7%)
**Parental Status**		
Has children	21 (95%)	11 (79%)
**IMPACT TOOLKIT revised version**		
No. who perpetrated at least one form of abuse within last 12 months		
Emotional abuse	14/19 (74%)	10/12 (83%)
Physical abuse	11/20 (55%)	9/14 (64%)
Sexual abuse	5/22 (23%)	3/14 (21%)
**Mean PHQ-9**^**a**^** score (SD)**	11.4 (7.7), 22	9.2 (7.6), 14
**Mean GAD-7**^**a**^** score (SD)**	9.5 (6.9), 22	8.3 (6.1), 14
**Mean PCL-5**^**a**^** score (SD)**	32.0 (21.7), 21	25.5 (22.1), 14
**Mean AUDIT**^**a**^** score (SD)**	11.6 (9.8), 20	8.1 (4.1), 12
**Mean DUDIT**^**a**^** score (SD)**	2.6 (4.2), 21	2.2 (3.0), 10
**Gambling problem (NODS-CLiP)**		
Yes	9/22 (41%)	5/14 (36%)

^a^High score is a worse outcome

**Table 5:** Baseline socio-demographic and clinical characteristics of female (ex)partners

Socio-demographics	Intervention *N* = 9^a^ n (%) or mean (SD)	Control *N* = 6 n (%) or mean (SD)
**Mean age (standard deviation, SD)**	44.1 (11.7)	42.8 (14.0)
**Sexuality**		
Heterosexual/Straight	8 (89%)	5 (83%)
**Ethnicity**		
White	6 (67%)	6 (100%)
Mixed / Multiple ethnic group	0	0
Asian or Asian British	0	0
Black /African / Caribbean / Black British)	0	0
**Religion**		
No religion	7 (78%)	4 (67%)
Christian	2 (22%)	2 (33%)
**Educational Qualifications**		
No formal qualifications	1 (11%)	0
Other qualifications	1 (11%)	0
O-levels, GC(S)Es, NVQs 1–3	2 (22%)	2 (33%)
A-levels	1 (17%)	1 (17%)
Degree or higher	3 (33%)	3 (50%)
**Employment Status**		
Employed	5 (56%)	3/5 (60%)
Looking after your home/family	2 (22%)	1/5 (20%)
Unable to work due to long term sickness	1 (11%)	0
Retired from paid work	0	1/5 (20%)
Other	1 (11%)	0
**Household Income**		
Up to £5000	1 (11%)	0
£5,000 up to £11,999	1 (11%)	0
£12,000 up to £21,999	1 (11%)	0
£22,000 up to £37,999	1 (11%)	0
£38,000 up to £71,999	2 (22%)	2 (33%)
£72,000 and above	2 (22%)	2 (33%)
Prefer not to say/do not know	1 (11%)	2 (33%)
**Parental Status**		
Has children	9 (100%)	4 (67%)
**IMPACT TOOLKIT revised version**		
No. who experienced at least one form of abuse within last 12 months		
Emotional abuse	6/8 (75%)	5/5 (100%)
Physical abuse	4/7 (57%)	6/6 (100%)
Sexual abuse	2/8 (25%)	1/4 (25%)
**Mean PHQ-9**^**b**^** score (SD)**	8.2 (4.6), 9	5.7 (4.2), 6
**Mean GAD-7**^**b**^** score (SD)**	7.3 (5.6), 9	5.0 (3.6), 6
**Mean PCL-5**^**b**^** score (SD)**	13.9 (10.7), 8	23.4 (14.2), 5
**Mean AUDIT**^**b**^** score (SD)**	8.2 (6.6), 6	6.7 (4.4), 6
**Mean DUDIT**^**b**^** score (SD)**	3.0 (3.0), 1	2.0 (2.0), 1
**Gambling problem (NODS-CLiP)**		
Yes	2/9 (22%)	1/6 (17%)

^a^2 female partners are the (ex)partners of one male participant

^b^High score is a worse outcome

### Data completeness

Table 6 reports the completeness of the nine-month outcome measures collected in the trial. Sixty-four percent of participants and 80% of (ex)partners completed the IMPACT toolkit at nine-months. Most other measures had similarly high completeness rates. Most men (33/36, 92%) were found on the local geographic police force database searched and it was assumed that the other men lived outside of the force area, or had no reports of incidents or crimes to police.

**Table 6:** Completion of outcome measures at nine-month follow-up

	**Intervention**	**Control**
	**n/N (%)**	**n/N (%)**
**IMPACT TOOLKIT revised version**		
Male participants	14/22 (63%)	9/14 (64%)
Female (ex)partners	6/9 (67%)	6/6 (100%)
**Mental health outcome measures (male participants)**		
PHQ-9	15/22 (68%)	9/14 (64%)
GAD-7	15/22 (68%)	9/14 (64%)
PCL-5	14/22 (64%)	9/14 (64%)
**Mental health outcome measures (female (ex)partners)**		
PHQ-9	6/9 (67%)	6/6 (100%)
GAD-7	6/9 (67%)	6/6 (100%)
PCL-5	6/9 (67%)	6/6 (100%)
**Drug/alcohol/gambling outcome measures (male participants**)		
AUDIT	12/22 (55%)	9/14 (64%)
DUDIT	12/22 (55%)	8/14 (57%)
NODS-CLiP	15/22 (68%)	9/14 (64%)
**Drug/alcohol/gambling outcome measures (female (ex)partners)**		
AUDIT	5/9 (56%)	6/6 (100%)
DUDIT	6/9 (67%)	6/6 (100%)
NODS-CLiP	6/9 (67%)	6/6 (100%)
**Health economic outcome measures (male participants)**		
EQ-5D-5L	9/22 (41%)	6/14 (43%)
ICECAP-A	9/22 (41%)	6/14 (43%)
**Health economic outcome measures (female (ex)partners)**		
EQ-5D-5L	1/9 (11%)	3/6 (50%)
ICECAP-A	1/9 (11%)	3/6 (50%)
**Kidscreen-10p (female (ex)partners)**		
** Kidscreen-10p**	3/9 (33%)	1/6 (17%)

### Intervention uptake and feasibility

Thirteen men (out of 22 allocated) attended over half the sessions (12 sessions or over) and nine men completed over three-quarters of the sessions (17 sessions). Five men withdrew either before attending a single session or after only one session. Two men were excluded by the programme delivery team (one before attending due to a high-risk police record, one after 11 sessions due to threatening behaviour). Six men attended additional sessions at their own request, repeating some sessions and attending more than the manualised 23. The median number of sessions attended (including non-attendees) was 13.0 (inter-quartile range 1 to 25, *n* = 22). The women’s safety workers supported 19 (ex)partners, which is nearly every intervention man’s (ex)partner. There was an average of 34.5 contacts (range 3 to 86) made per woman (home visits, texts, phone calls and accompanying them to court). It was feasible to set up the intervention delivery and train the delivery team in a timely manner. All of the team attended the four-day training. There were some delays in setting up a full and appropriate supervisory structure.

### Towards a fidelity framework

We distinguished between programme integrity (whether staff delivering the intervention received timely and appropriate clinical and management supervision, whether the service was working within a multi-agency framework, and whether a positive programme ethos was present) and fidelity to the intervention model (whether the sessions followed the programme manual while allowing appropriate flexibility and if delivery style and principles of the programme were upheld). In the definitive trial, assessment of programme integrity will be partly met through the delivery organisation’s accreditation process with Respect and a proportion of video or in-person observations of the groups. Fidelity to the model will be assessed by developing core session objectives and video/in-person observations, please also see group observation template (Additional file 3).

### Economic perspective, quality of life and resource-use data

Given the multi-agency involvement as well as the multi-system impact of this intervention, the economic analysis was conducted from a health perspective and a wider societal perspective. This wider perspective included judicial system involvement, personal non-heath costs associated with IPV and time missed in education for children.

We employed an additional quality of life measure, the ICECAP-A (ICEpop CAPability for Adults, [49, 50]) to capture non-health-specific benefits of the intervention for both male participants and their (ex)partners. This measure was introduced in the nine-month questionnaire following consultation with our PPI contributors, who recognised the relevance of the dimensions around stability (feeling settled and secure), attachment, autonomy, achievement and enjoyment. To capture wider costs, we developed resource-use questions which included items on criminal justice system resource, personal accommodation costs and specific third-sector services for adults, child social services and education for any dependent children, as well as standard items on use of health services. Analysis of resource-use completion highlighted the importance of general practice contacts, psychological and talking therapies, social care and third sector agencies as likely cost drivers. Questions on accommodation were poorly answered and associated costs were variable, so were removed from the nine-month questionnaire.

### Intervention acceptability

The intervention was considered acceptable by most participants (perpetrators and (ex)partners), and delivery staff. The findings concerning the acceptability of the intervention divided into two broad themes: the benefits of involvement in the intervention and the difficulties of involvement in the intervention. In interviews, men who completed all or most of the programme gave positive feedback about the trial and reported beneficial changes in their own behaviour. Men reported the peer support element of the groups to be helpful and some said they felt committed to the group because of their shared purpose and journey. One man said “I think frustration and anger is quite isolating. It’s not something you talk about with your mates. And you go into a room with other people, and instantly you’ve got support…in their sharing, and in your own sharing, that’s where the work gets done”. Tools for reducing abusive behaviours and reinforcing general life skills were also beneficial. Several men who disengaged with the programme said their behaviour and relationship had improved, and they no longer needed the group. One man said that the group made him feel bad about himself. In interviews women were largely positive about their (ex)partners’ attendance in the group intervention. One woman said “He’s quicker to try to resolve things. Whereas, before it could carry on for days or even weeks – or not be resolved”. All women linked to intervention men who were offered a women’s support worker accepted this help, and feedback was positive. The majority of these women were not previously in receipt of support from domestic abuse specialists. The women’s safety worker also reported getting some additional and positive feedback from the women that she had been working with including older children in the family noticing differences in their father’s behaviour, men being more reflective, and men’s ‘tone of voice’ changing. From interview feedback with staff delivering the men’s group programme, they found it broadly acceptable although some group facilitators had found swapping to a different and unfamiliar manual challenging. At an end-of-pilot-trial focus group the staff had minor suggestions for improving the manual such as requests for some additional material to cover stalking and harassment, and how to safely manage specific seasonal occasions, such as Christmas. The training was well received by staff. In recognition of wider developments in the field of domestic abuse perpetrator response [51] the research team suggested that programme elements related to trauma were more clearly identified in the manual including some additional exercises.

### Involvement of (ex)partners

Contact details for (ex)partners were taken from all but one male participant where there were safety concerns. Contact was attempted by the research team with all (ex)partners, but only fourteen of the men’s (14/36, 39%) (ex)partners were recruited into the trial. In interviews women often said they joined the trial for altruistic reasons to help others in similar situations. Some said they hoped their (ex)partner would change behaviour if he was involved in the trial. Some men misleadingly reported their involvement in the trial to their (ex)partners. To reduce the risks of wrongful reporting we informed all (ex)partners about their (ex)partner’s involvement in the trial and allocation.

### Improving retention

Significant time was dedicated to following-up questionnaires, via calls, text messages and emails. Completing questionnaires over the phone improved retention. A £5 shopping voucher was offered for each completed baseline questionnaire, which increased at each follow-up (to a total of £50). Retention-related tasks for (ex)partners was sometimes delayed because of research team capacity. The follow-up rate was lower in the control group. When men in the intervention prematurely left the DAPP, they often disengaged with the research, as did their (ex)partners. The PPI groups gave advice about improving retention and, following their advice, we shortened the questionnaire.

#### Support and supervision for intervention facilitators

In normal practice, support and supervision in DAPPs is covered by the Respect accreditation process in line with their standards. As the delivery organisation was not due for accreditation renewal the research team engaged in some monitoring to help inform implementation, such as asking about case management and clinical supervision arrangements. It was found that an independent monitoring role was a crucial element to ensure good service delivery, and accreditation was planned for the definitive trial.

#### Primary outcome for the definitive trial

There are potential biases to participants’ self-reporting the level, frequency and impact of abuse. Male abusers may minimise their abusive behaviour whereas (ex)partners may feel coerced to do likewise, either directly by their partner, or from fear of potential action by social services (such as removing their children). We concluded that although the female (ex)partners’ reporting gave crucial credibility, the primary outcome should be reported by the male participants because they were the randomised participant and not every (ex)partner may have been involved. As the revised IMPACT toolkit [37] had not previously been assessed in a trial, been validated or a scoring system devised, the primary outcome for the definitive trial will be the modified Abusive Behaviour Inventory (ABI) [52]. We excluded a question about “spanking” as recommended by Postmus and colleagues [53], and rephrased from “bad parent” to “bad person”, as not all participants may be parents. We also included a “not applicable” response to questions about children and driving.

#### Serious Adverse Events and safety considerations

Two men were withdrawn from the intervention group and the research due to safety concerns (Fig. 1). There were three serious adverse events reported, two of which were unrelated to the trial, and one potentially related to the trial. Unforeseen risks identified included a male participant who gave a false name, lied to his partner (not involved in the trial) about his randomisation, and injured her. Strategies used to address these risks were to request men to give proof of identity at recruitment and providing an information sheet for partners stating the man’s allocation. (Ex)partners who were deemed to be at increased risk if they participated in the trial were not approached (Fig. 3).

## Discussion

We were able to recruit male participants and their female (ex)partners to a pilot trial of a community-based voluntary DAPP. Referral routes took time to establish and there was some resistance to referral within social work teams given that a third of men would not be allocated to receive the intervention due to randomisation. The intervention, manual and training were acceptable to facilitators and delivery staff with some slight modifications, such as additional exercises on stalking and harassment and Christmas safety planning. The intervention was acceptable to participants. By this we mean that the perceived benefits of the intervention outweighed any challenges encountered by the majority of those we interviewed and from whom we received feedback. Male participants reported that they had benefited from the group programme and female participants reported positive experiences of the support they received and felt there were positive and noticeable changes in their partner’s behaviour. Some men in the control group reported that they felt they had changed their behaviour as a result of completing the questionnaires, as it enabled them to reflect on their recent behaviour and reminded them of their desire to make positive changes. This is known as measurement reactivity which is an issue in designing pragmatic trials and needs to be considered in sample size calculations [54]. Retention and completeness of data were reasonable in both randomised groups.

There were some safety benefits to (ex)partners and their families in being more visible to professionals, as a significant proportion said that they had not previously received help from specialist domestic abuse agencies. There were also risks associated with the trial. The intervention may have supported female partners to leave their partners, and abusers are often at their most dangerous when they feel they have nothing left to lose [55, 56]. Alternatively, female partners may have stayed in relationships because they believed that the intervention would be effective. Male intervention participants were more likely to be challenged in their beliefs, and increased realisation and responsibility for previous abuses may have caused considerable mental distress and feelings of shame, causing increased risk of depression, self-harm and suicide, or femicide-suicide [57, 58]. Researchers could miss serious abuse occurring for partners of control participants if they did not report information, as reporting and multiagency working increased the information known about partners of intervention participants. Within the intervention itself there were several core safety elements to help protect participants such as women’s safety workers conducting regular risk assessments with the partners, information sharing between delivery staff, and referrals to other agencies when male or female participants disclosed information that indicated risk escalation. Women’s safety workers would also always specifically discuss the possibility of false hopes being raised with all female partners that they supported alongside other ongoing safety planning. Going forward, to try and mitigate against any female partners in the control arm also having false hopes of behaviour change through trial involvement (as opposed to intervention involvement) the research team decided to contact women and explain the study earlier (before the man’s assessment) so that the research team could clarify what involvement might mean. While recognising an imbalance of information on control and intervention arm participants that could help assess and manage risks the research team monitored and acted on any verbal information given during contact or information written in the questionnaires. For example, following our safety protocol the research team checked all returning questionnaires and tried to contact all participants who indicated suicide ideation on the PHQ-9 [38] for a conversation and did follow up calls to their general practitioner as needed. The research team also followed up or took actions on other ‘trigger’ information received such as disclosure of information about hospitalisation or other specific written or verbal comments of concern. Discussion within the research team followed by discussion with the team senior researcher and clinician as needed was the standard procedure for all areas of concern. Overall, we balanced the risks of participation or non-participation against the knowledge that DAPPs are being commissioned in the UK and internationally without evidence of their effectiveness.

### Limitations

There were delays to starting the intervention as a minimum number of men were needed to start a viable group. The need for a relapse prevention group was initially overlooked as it was not part of the Respect guidance for accredited DAPPs but we recognised this was a key element of the intervention. It was not possible to blind either the participants or the research team to the allocation. Recruitment and retention of men was prioritised over (ex)partners and there were risks of information leaks when the same researcher recruited both male participants and their female (ex)partner. The measure chosen as the primary outcome for the definitive trial was not evaluated in this pilot trial.

### Comparison with existing literature

This randomised pilot and feasibility trial sought to overcome many of the reported methodological shortcomings associated with DAPP evaluations such as non-randomised design, a predominance of court-mandated populations, over-reliance on police incident report data, high attrition and short follow-ups [10, 25, 28, 32]. We have shown that it is feasible to recruit men outside of court-mandated populations from organisations, or by self-referral, after adaptations to our recruitment procedures and randomisation ratio. We had high rates of self-completed participant reported data (64% of male participants, 80% of (ex)partners completed the IMPACT toolkit at nine-months), with high rates of retention (67% of male participants, 80% of (ex)partners) over nine-months of follow-up. The intervention was seen as acceptable by most of the participants and who reported similar benefits to other studies. [7, 17–19]. Although there is little consensus in the literature about what programme integrity means in relation to DAPPs, our framework for assessing programme integrity and fidelity to the intervention builds on the work of Bowen and Gilchrist [59], Phillips [60], Kelly and Westmarland [19] and is in line with Respect standards (4th edition [61]). We have been careful not to be overly reliant on an idea of adherence to programme manuals to ‘prove’ efficacy as we recognise facilitators must also draw continuously on their professional judgement and expertise to make sure that they are working flexibly and responsively with the material that is brought by participants into group work.

## Conclusions

This pilot and feasibility trial has shown that a definitive trial investigating the effectiveness and cost-effectiveness of a DAPP is feasible and desirable. Inclusion and exclusion criteria were largely suitable and only needed slight refinements. For example, we clarified that recent contact (within the last year, or about to have contact for men coming out of prison) was needed with an abusive partner and a man must not have attended a group domestic abuse intervention that was any longer than eight weeks. To improve referrals rates and engagement we changed the randomisation ratio from 1:1 to 2:1. Research specific safety considerations to be continued included: contacting women prior to men’s assessments; requiring proof of identity at assessment for men; sending statements of trial allocation to women after assessments; close monitoring of questionnaire responses; and being aware of any written or verbal comments made during contact that might indicate escalating risk. A dedicated researcher would be needed to improve recruitment and retention of women, to minimise the (perceived or actual) risk of information leaks between participants. Consent to link data on trial outcomes with police records is needed for both groups, as only intervention men had given consent in this pilot. The candidate primary outcome in the pilot trial was not suitable, and a primary outcome was chosen that was untested in this pilot trial. After review of the intervention manual, there were only slight modifications such as additional exercises to address stalking and harassment, trauma responses and planning for safely managing peak times of stress such as the Christmas period. A reduced and simplified accreditation process would be needed to support intervention integrity and fidelity. For more information on the full trial please see the published full trial protocol [62].

### Supplementary Information

**Supplementary Material 1.**

**Supplementary Material 2.**

**Supplementary Material 3.**

## Data Availability

The datasets (quantitative) generated and analysed during the current study are not publicly available due to the highly sensitive nature of the data which might place victims/survivors of domestic abuse at further risk but are available to bona fide researchers on reasonable request at the University of Bristol Research Data repository at https://doi.org/10.5523/bris.fven0hg6fpr629x7eg2nf4hb3. The datasets (qualitative and police) generated and analysed during the current study are not publicly available due to the highly sensitive nature of the data which might place victims/survivors of domestic abuse at further risk.

## Abbreviations

ABI: Abusive Behaviour Inventory
AUDIT: Alcohol Use Disorders Identification Test
CBT: Cognitive Behavioural Therapy
CJS: Criminal Justice System
CONSORT: Consolidated Standards of Reporting Trials
DAPP: Domestic Abuse Perpetrator Programme
DUDIT: Drug Use Disorders Identification Test
HND: Higher National Diploma
GAD-7: Generalised Anxiety Disorder Assessment
GC(S)E: General Certificate of Secondary Education
ICECAP-A: ICEpop CAPability measure for Adults
IPV: Intimate partner violence
MARAC: Multi-Agency Risk Assessment Conference
NODS-CLiP: NORC Diagnostic Screen for Gambling Disorders—Control, Lying, and Preoccupation
NVQ: National Vocational Qualification
PCL-5: Post-traumatic stress disorder CheckList for DSM-5
PHQ-9: Patient Health Questionnaire
PPI: Public and patient involvement
REC: Research ethics committee
RPG: Relapse prevention group
SD: Standard deviation
WHO: World Health Organisation
