# Supplementary material for: Randomised pilot and feasibility trial of a group intervention for men who perpetrate intimate partner violence against women
Source: BMC Public Health. 2024 Apr 27;24:1183. doi: 10.1186/s12889-024-18640-5 (PMC11055266; doi:10.1186/s12889-024-18640-5)
Supplement: Supplementary file 1 — Supplementary Material 1. [file 12889_2024_18640_MOESM1_ESM.pdf]

Additional file 1. Logic model

Problem: there is limited evidence that domestic violence perpetrator programmes are effective

Goal: to increase safety & reduce abuse for partners & ex partners of DV perpetrators

Populations

Population: male DVA perpetrators referred from social services, police, health & self referred

Population: linked female DVA ex/partners

Population: linked children of DVA perpetrators & ex/partners

Theory & assumptions

Gendered learning, trauma and lack of accountability lead to: unrealistic & unequal expectations of women; low tolerance of vulnerability; attachment disorders; raw & sore points; poor self regulation; low partner empathy; poor conflict resolution skills; poor parenting skills; selfish / blaming & hostile stance; low motivation to change.

There will be interpersonal influences on ability to change such as past trauma [1], mental health [2], autistic spectrum disorder [3], substance misuse [4], presence of children, relationship status, age, education, housing, nationality, ethnicity, religion [5]

Intervention

Group work [6], individual sessions [7], relapse prevention group [8], interagency work [9] & partner work [10]

Core components: accountability; mindfulness; trauma insight; sexual respect; anger management; CBT; emotional regulation; empathy building; conflict resolution; parenting skills; narrative therapy.

Group work would include the overall ethos, interaction style & positive group dynamic with skilled facilitators. Educational & skills practice elements. Men will be encouraged to engage, recognise warning signs, 'straight talk' about what they have done, develop a critical self awareness of attitudes, beliefs & expectations.

Case work involving safety planning & advocacy for female ex/partners [10]

Proximal outcomes

Abusive behaviour ↓ [11]
Individual functioning↑:
Emotional regulation ↑, anger management ↑ [12].
Interpersonal skills: problem solving, conflict resolution, empathy ↑, communication & parenting skills ↑, listening ↑ [13], sexual respect ↑ [14]
Motivation to change ↑ [15]
Service use ↑[16]

Safety ↑, experience of abusive behaviour ↓ [17]
Individual functioning↑[12]:
Service use ↑[16]

Safety ↑[17]
Individual functioning ↑[18]

Long term outcomes

Wellbeing↑[19], depression/anxiety/PTSD ↓↑ [20]
Cost savings associated with perpetration [21]
Breaking cycle of violence

Safety & wellbeing ↑ [22] depression/anxiety/ PTSD↓ [20]
Cost savings associated with perpetration [21]
Breaking cycle of violence

Safety & wellbeing ↑ [17,22]
Breaking cycle of violence

Evaluation key for logic model

	Variable		Method of evaluation or measurement
1.	Past trauma	1.	Self-report on childhood experiences. PC-PTSD-5. Interviews.
2.	Mental health	2.	Self-report PHQ-9, GAD-7, PC-PTSD-5. ICE-CAP, EQ5-D & SF-12 Interviews.
3.	Autistic spectrum disorder	3.	Perpetrator only self-report AQ-10. Interviews.
4.	Substance misuse	4.	Self-report AUDIT C, DUDIT. Interviews.
5.	Sociodemographic characteristics: presence of children, age, education, housing, nationality, ethnicity, religion	5.	Self-report on children, relationship status, age, housing, nationality, employment status, income, ethnicity, religion.
6.	Group intervention	6.	Group work would include the overall ethos, interaction style & positive group dynamic with skilled facilitators. Educational & skills practice elements. Men will be encouraged to engage, recognise warning signs, 'straight talk' about what they have done, develop a critical self awareness of attitudes, beliefs & expectations. Fidelity analysis to principles of programme & (basic) adherence to manual from sample of videoed and observed sessions + implementation support from Respect
7.	1-to-1 support	7.	Number and type of 1-to-1 support, referrals made, court reports, agency contact. Interviews.
8.	Relapse prevention group (RPG)	8.	Number of RPG sessions attended. Interviews.
9.	Interagency work	9.	Reports from DVPP coordinator & facilitators, court reports, SAEs. Interviews with DVPP staff.
10.	Support from women's safety worker	10.	Number and type of contacts with women's safety worker, court reports, SAEs. Interviews.
11.	Abusive behaviour	11.	Perpetrator self-report Abusive Behaviour Inventory + impact measures from IMPACT TOOLKIT + Propensity for Abusiveness Scale + adapted-IPVRAS. Police reports + SAEs. Reports from DVPP coordinator & facilitators. Interviews.
12.	(Perpetrator) Individual functioning: Emotional regulation, anger management	12.	Perpetrator self-report. PHQ-9, GAD-7, PC-PTSD-5, ICE-CAP & SF-12 (emotional regulation), Propensity for Abusiveness Scale (anger). Interviews.
13.	Interpersonal skills: problem solving, conflict resolution, empathy, communication & parenting skills, listening	13.	Adapted-IPVRAS (self reflection), Adapted communications patterns questionnaire- short form (conflict resolution, listening, empathy, listening, self reflection), Propensity for Abusiveness Scale (self reflection). + impact measures on parenting from IMPACT TOOLKIT. Reports from DVPP coordinator and facilitators. Referrals made, court reports, agency contact. Interviews.
14.	Sexual respect	14.	Abusive Behaviour Inventory (sexual items) – perpetrator & Victim. Interviews.
15.	Motivation to change	15.	Adapted-IPVRAS (responsibility attribution). Initial assessment from DVPP coordinator. Reports from DVPP coordinator and facilitators. Attendance and completion of DVPP. RPG attendance. DNA information. Questionnaire completion. Interviews.
16.	Service use	16.	Self report use of health and social services, medication use, housing, employment and benefits, use of children's services. EQ5D, , ICE-CAP, CHU-9D + impact measures from IMPACT TOOLKIT. Criminal justice system costs through police reports (perpetrator only). Referrals made, court reports, agency contact. SAEs, GP records. Interviews.
17.	Victim safety & experience of abusive behaviour	17.	Victim self-report ABI, SF12(v2), EQ5D, PHQ-9, GAD-7, PC-PTSD-5. AUDIT C, DUDIT. SAEs . Interviews.
18.	Child individual functioning	18.	Parent (victim) report ICECAP-A . Interviews with victim. Impact measures from IMPACT TOOLKIT
19.	Perpetrator wellbeing	19.	Self-report SF12(v2) EQ5D, PHQ-9, GAD-7, PC-PTSD-5. AUDIT C, DUDIT. Interviews.
20.	Depression/anxiety/PTSD	20.	PHQ-9, GAD-7, PC-PTSD-5. SF12(v2) EQ5D, ICE-CAP. SAEs. Interviews.
21.	Cost effectiveness	21.	Cost effectiveness analysis accounting for intervention costs, criminal justice costs and self-report service costs.
22.	Victim safety & wellbeing	22.	Victim self-report SF12(v2) EQ5D, ICE-CAP, PHQ-9, GAD-7, PC-PTSD-5. AUDIT C, DUDIT. SAEs . Interviews.
