# Supplementary material for: Randomised pilot and feasibility trial of a group intervention for men who perpetrate intimate partner violence against women
Source: BMC Public Health. 2024 Apr 27;24:1183. doi: 10.1186/s12889-024-18640-5 (PMC11055266; doi:10.1186/s12889-024-18640-5)
Supplement: Supplementary file 2 — Supplementary Material 2. [file 12889_2024_18640_MOESM2_ESM.docx]

**Additional file 2. Coding framework**

| **1^st^ level** | **2^nd^ level** | **3^rd^ level** |
| --- | --- | --- |
| 1. Abuse | A1 Types of abuse |  |
|  | A2 Causes of abuse | A2.1 What caused ‘your’ domestic violence and abuse (DVA)? |
|  | A3 Understanding of abuse |  |
|  | A4 Stigma and hidden nature of abuse |  |
|  | A5 Childhood experiences |  |
|  | A6 Impact of abuse |  |
| 1. Perpetrator DVA help-seeking | B1 Trigger for help-seeking |  |
|  | B2 Explanation of referral method |  |
|  | B3 Types of DVA help-seeking |  |
|  | B4 Barriers to help-seeking (male) |  |
|  | B5 Facilitators to help-seeking (male) |  |
|  | B6 Missed opportunities for perpetrator help (male defined) |  |
| 1. Partner DVA help-seeking | C1 Partner explanation of trigger |  |
|  | C2 Partner explanation of barriers to male help-seeking |  |
|  | C3 Partner explanation of facilitators to male help-seeking |  |
|  | C4 Partner’s own help-seeking |  |
|  | C5 Barriers and facilitators for partner help-seeking |  |
|  | C6 Missed opportunities for perpetrator help (female defined) |  |
|  | C7 Missed opportunities for victim help (female defined) |  |
| 1. Motivation | D1 Perpetrator description of motivation to join trial |  |
|  | D2 Perpetrator description of motivation to remain engaged on trial |  |
|  | D3 Female description of perpetrator motivation to join study |  |
|  | D4 Female description of perpetrator motivation to remain engaged |  |
| 1. Other support or agencies | E1 Anger-management help-seeking |  |
|  | E2 Therapeutic help-seeking |  |
|  | E3 GP help-seeking |  |
|  | E4 Drug / alcohol support |  |
|  | E5 Other support |  |
|  | E6 Mental health support |  |
|  | E7 Police |  |
|  | E8 Social services |  |
| 1. Relationships | F1 Relationship with abused (ex) partner | F1.1 Pressures in relationship |
|  |  | F1.2 Relationship changes |
|  |  | F1.3 Hopes for relationship |
|  | F2 Previous relationships |  |
|  | F3 Relationship with parents |  |
|  | F4 Relationship with children |  |
|  | F5 Social and wider family networks |  |
|  | F6 New intimate partner relationship |  |
| 1. Behaviour change | G1 Perpetrator reported change in abuse (what) |  |
|  | G2 Partner reported change in abuse (what) |  |
|  | G3 Perpetrator explanation for change |  |
|  | G4 Partner explanation for change |  |
|  | G5 Strategies for change |  |
|  | G6 Sustainability of change – perpetrator thoughts |  |
|  | G7 Sustainability of change – perpetrator thoughts |  |
| 1. Identity | H1 Fatherhood or motherhood |  |
|  | H2 Gendered perspectives | H2.1 Masculinities |
|  |  | H2.2 Feminism / femininities |
|  | H3 Victim / perpetrator |  |
|  | H4 Childlike identity |  |
| 1. Accounts of abuse | I1 Denial |  |
|  | I2 Blaming |  |
|  | I3 Minimising |  |
|  | I4 Justification |  |
|  | I5 Taking responsibility |  |
|  | I6 Shifting language about abuse |  |
|  | I7 Self pity |  |
| 1. Trial | J1 General trial-related |  |
|  | J2 First assessment meeting |  |
|  | J3 Questionnaires | J3.1 Easy to follow |
|  |  | J3.2 Length |
|  |  | J3.3 Relevance or content |
|  |  | J3.4 Reasons for completing |
|  |  | J3.5 Reaction to questions |
|  | J4 Randomisation | J4.1 Understanding of randomisation |
|  |  | J4.2 Reaction to allocation |
|  | J5 Impact on relationship of being in the trial |  |
|  | J6 Signposting |  |
|  | J7 Perpetrator hopes for joining trial |  |
|  | J8 Partner hopes for trial |  |
|  | J9 Wider impacts of intervention |  |
| 1. Intervention experiences of group | K1 Initial feelings |  |
|  | K2 Other men in group | K2.1 First meeting with other men |
|  |  | K2.2 Ongoing relationship with other men |
|  |  | K2.3 Challenged by or challenging other men |
|  | K3 Group Facilitators |  |
|  | K4 Group Coordinator |  |
|  | K5 Group dynamics | K5.1 Benefits or strengths of group |
|  |  | K5.2 Negatives or weaknesses of group |
|  | K6 Key learning from programme | K6.1 Tools |
|  |  | K6.2 Most/least valuable parts of programme |
|  | K7 1:1 sessions |  |
|  | K8 Overall challenges of attending |  |
|  | K9 Next steps (RPG?) |  |
|  | K10 Epiphanal moments from group |  |
| 1. Mental Health | L1 Participant’s own mental health (men) | L1.1 Mental health affecting relationship |
|  | L2 Partner’s mental health (women) |  |
| 1. Women’s support worker | M1 Reasons for accepting or rejecting support |  |
|  | M2 Type of support offered |  |
|  | M3 Comments on support |  |
|  | M4 Control women and DVA support |  |
| 1. Other | N1 ‘Truth’-talk |  |
|  | N2 External changes during programme period |  |
| Z. Fabulous quotes |  |  |
| PA. Practitioner perspectives on DVPP | PA1. DVPP Challenges | PA1.1 Promotion  PA1.2 Recruitment (acceptance/ resistance) |
|  | PA2. Attitudes towards effectiveness of domestic violence perpetrator programmes (DVPPs) |  |
|  | PA3. Feedback on exclusion criteria |  |
|  | PA4. Key Resources for DVPP delivery | A6.1 Power and Control Wheel  A6.2 RESPECT RIC |
|  | PA5. Fidelity to programme |  |
|  | PA6. Understanding of purpose of programme |  |
| PB. Practitioner perspectives on, and experiences of, study | PB1. Practitioner understanding of, and concerns about, study |  |
|  | PB2. Impact of study on participants |  |
|  | PB3. Evidencing / evidence base |  |
|  | PB4. Fairness/ ethics (RE: control group not receiving support) |  |
|  | PB5. Differences in roles of practitioners and researchers |  |
|  |  |  |
|  |  |  |
| PC. DVPP Processes | PC1. Assessment process |  |
|  | PC2. Information sharing |  |
|  |  |  |
| PD. Participant engagement (in programme) | PD1. Levels of engagement |  |
|  | PD2. Perpetrator motivations for engagement/ non-engagement (e.g. tick-box, performance etc.) |  |
|  |  |  |
|  |  |  |
|  |  |  |
|  |  |  |
| PE. Participant Motivation (practitioner perspectives) | PE1. Help-seeking |  |
|  | PE2. Pressures exerted from referring service  (compelled by referral agency – e.g. Soc Services) |  |
|  | PE3. Fear of loss (of partner/ of control?) |  |
|  |  |  |
| PF. Alternative support for perpetrator of DVA/ DVPP Alternatives |  |  |
