# Supplementary material for: Randomised pilot and feasibility trial of a group intervention for men who perpetrate intimate partner violence against women
Source: BMC Public Health. 2024 Apr 27;24:1183. doi: 10.1186/s12889-024-18640-5 (PMC11055266; doi:10.1186/s12889-024-18640-5)
Supplement: Supplementary file 3 — Supplementary Material 3. [file 12889_2024_18640_MOESM3_ESM.docx]

**Additional file 3. Group observation template***

*Name of organisation: Date of group session:*

*File name: Facilitators:*

*Assessor 1: Date & time of review:*

*Assessor 2 (if double coded):*

| **Key areas of practice to identify related to service integrity and programme fidelity** | **√ or x** | **Notes and examples (good and poor practice)** |
| --- | --- | --- |
| **Clear analysis** of domestic violence and abuse including purpose  (Are facilitators picking up on the participant’s intention and goals of abusive behaviours and working on this?) |  |  |
| Diminished **minimising, denying** and blaming  (Are facilitators regularly picking participants up on these behaviours and is it clear the facilitators are really listening?) |  |  |
| Promoted taking **responsibility** for own behaviour and effects |  |  |
| Promoted **questioning** attitudes **of gender-based entitlement** |  |  |
| Facilitated **respectful group** process, including of ethnicity, faith etc.  (Do facilitators ensure all participants get an equal opportunity to speak at check-ins and throughout?) |  |  |
| Promoted **safe, child-focused parenting** |  |  |
| Promotes abilities to have **respectful relationships** |  |  |
| Increased understanding and empathy for **effects on victim/survivors** |  |  |
| Modelled and promoted **gender equality** |  |  |
| **Safety of victims/survivors** promoted as priority  (Do facilitators help participants to understand the consequence of their actions on their partners?) |  |  |
| Promoted **reflective thinking, participation** and dialogue  (Do facilitators allow opportunity for discussion, do they ask open questions, make relevant associations, link abusive behaviours to self-beliefs and point out cognitive dissonance? Also core to a motivational interviewing approach). |  |  |
| Provided ample **opportunity to present material**  (Do facilitators give enough time to the different aspects of the session? Are check-ins too long, or purposeful, focused and concise? Is there enough time to cover an exercise or teach a new skill?) |  |  |
| Adhered to **group rules** |  |  |
| Are tools used **exploratively, collaboratively, and sensitively or rigidly?** |  |  |
| **Working alliance** (building bond, shared understanding and tasks) **and engagement** (between participants and facilitators) |  |  |
| **Facilitator delivery style and stance**  (Describe facilitators style and stance. Do they facilitate conversations or ‘teach’? Do facilitators step in and out of the conversation as needed? Is the style of co-working with the other facilitator collaborative?) |  |  |
| **Flexibility between process and content**  (Is there the right balance allowing enough flexibility to be process driven rather than content driven?) |  |  |
| **Trauma informed** approach  (Do the facilitators use any tools or techniques which recognise or help those with trauma?) |  |  |
| Covered **core objectives** of session? |  |  |
| *If double scored were there any big differences of opinion? If yes note here:* | | |

*Modified template based on an observation checklist developed by Respect for use when assessing treatment management, with kind permission.
